# Wavefront Shaping Concepts for Application in Optical Coherence Tomography—A Review

**DOI:** 10.3390/s20247044

**Published:** 2020-12-09

**Authors:** Jonas Kanngiesser, Bernhard Roth

**Affiliations:** 1 Hannoversches Zentrum für Optische Technologien, Leibniz Universität Hannover, Nienburger Straße 17, D-30167 Hannover, Germany; jonas.kanngiesser@hot.uni-hannover.de; 2 Cluster of Excellence PhoenixD (Photonics, Optics, and Engineering–Innovation Across Disciplines), D-30167 Hannover, Germany

**Keywords:** optical coherence tomography, wavefront shaping, adaptive optics, signal enhancement, non-invasive diagnostics, in-vivo imaging, scattering media

## Abstract

Optical coherence tomography (OCT) enables three-dimensional imaging with resolution on the micrometer scale. The technique relies on the time-of-flight gated detection of light scattered from a sample and has received enormous interest in applications as versatile as non-destructive testing, metrology and non-invasive medical diagnostics. However, in strongly scattering media such as biological tissue, the penetration depth and imaging resolution are limited. Combining OCT imaging with wavefront shaping approaches significantly leverages the capabilities of the technique by controlling the scattered light field through manipulation of the field incident on the sample. This article reviews the main concepts developed so far in the field and discusses the latest results achieved with a focus on signal enhancement and imaging.

## 1. Introduction

Optical coherence tomography (OCT) is closely related to white light and low coherence interferometry as well as to optical coherence domain reflectometry which was originally developed to locate defects in optical fibers [1,2] and soon proved to be feasible for biomedical imaging [3,4]. The group of Fujimoto presented the first OCT system for the imaging of ex-vivo biological tissue in 1991 [5]. The first in-vivo imaging applications were reported in 1993 independently by Fercher et al. [6] and by Swanson et al. [7]. Here, we restrict ourselves to a brief description of the main OCT concepts as required for a proper understanding of the wavefront shaping implementations. For a detailed discussion, we refer to the references, as indicated at the appropriate positions in the text.

OCT is based on the interference of broadband light, which allows one to determine the time-of-flight or the optical path length distribution of the electromagnetic wave reflected at a sample. Typically, OCT systems use point-wise sample illumination, i.e., the beam is focused at the sample similar to confocal microscopy. The reflected beam is collected by the imaging optics and superimposed with a static reference beam which has a well-known optical path length.

**Figure 1:**
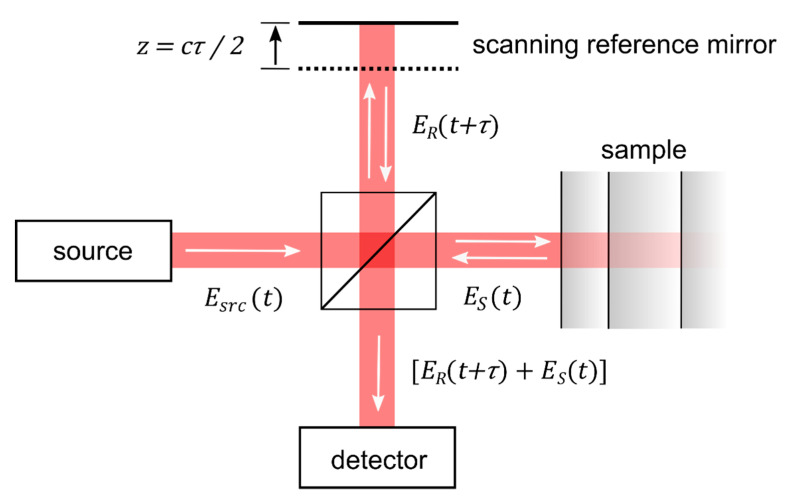
Principle of optical coherence tomography. The technique is comparable to a Michelson interferometer with the sample placed at one interferometer arm. Scanning the length of the reference arm allows one to determine the time-of-flight of the beam which is backscattered from the sample. Variables are as in the text. Image from [8], with permission.

The principle of optical coherence tomography is well-understood by considering a Michelson interferometer with the sample placed in one interferometer arm (Figure 1). The approach represents one important class of OCT systems, namely time domain OCT (TD-OCT).

The beam incident in the interferometer is described by its electric field E_src_, which is coupled to the magnetic field through Maxwell’s equations. The incident field E_src_ is divided at the beam splitter, reflected at the two interferometer arms, recombined and detected. The field in the plane of the detector, thus, reads *E_R_*(*t*) + *E_S_*(*t*), where *E_R_* describes the field returned from the reference arm and *E_S_* the field returned from the sample. Displacing the reference mirror by the distance z increases the length of the reference arm and introduces an additional temporal delay *τ* = 2 z/c to the reflected reference beam where c is the speed of light. At the detector, the intensity of the superimposed beams of a TD-OCT setup *I^TD^* reads [9,10,11,12]:

(1) $$I^{TD}\left(\tau \right)\propto {\left\langle |E_{R}\left(t+\tau \right)+E_{S}\left(t\right)\right|}^{2}\rangle \;={\left\langle |E_{R}\left(t\right)\right|}^{2}\rangle +{\left\langle |E_{S}\left(t\right)\right|}^{2}\rangle +2R\left\{\Gamma_{RS}\left(\tau \right)\right\}$$

Here, *E_R_* and *E_S_* stand for the field returned from the reference and sample arm, respectively, *t* denotes the time, *τ* the above temporal delay, the brackets stand for the time averaged values and $ℜ\left\{\Gamma_{RS}\left(\tau \right)\right\}$ for the real-part of the cross-correlation term (see below). The first two terms in the second line of Equation (1) correspond to the intensity returned from the reference and sample beam, respectively. The third term yields the interference of the two beams and describes the real part of the field cross-correlation $\Gamma_{RS}\left(\tau \right)=\;\langle E_{R}\left(t+\tau \right)E_{S}^{*}\left(t\right)\rangle$, which is also termed cross coherence or mutual coherence function [9,10,13] (* denotes the complex conjugated quantity). Thus, TD-OCT essentially captures the intensity of the superimposed reference and sample beam at a point detector while scanning the length z or the temporal delay at the reference beam. A full scan yields a single depth scan, also known as A-scan, at the point at which the sample is illuminated. Laterally scanning the sample beam in one direction yields a cross-sectional image perpendicular to the sample surface, i.e., a B-scan, while additional scanning the second lateral direction yields a C-Scan, i.e., a volumetric (3D) image. Most practical OCT systems are based on fiber-optic interferometers. A set of scanning mirrors allows one to scan the sample beam laterally to enable cross-sectional and volumetric imaging.

An important aspect is the depth of field (DOF) which describes the axial range at which the sample beam remains tightly focused. The DOF depends on the Rayleigh length of the sample beam and, hence, becomes smaller in case the sample beam is more tightly focused. With most OCT systems, low NA objective lenses are used to achieve a large DOF. Axial imaging is performed by scanning the reference mirror over distances of several millimetres. A large DOF, hence, is required to maintain a comparable lateral resolution and sensitivity at the full axial field of view (FOV) of the OCT system. In case a low NA objective lens is used, the impact of the imaging optics on the axial sensitivity of the OCT system can be neglected. With regard to resolution, in contrast to conventional optical imaging approaches, the lateral and axial resolution of OCT systems can be tuned independently.

TD-OCT systems acquire the cross-correlation by scanning the length (or the temporal delay) of the reference beam and by capturing the intensity of the superimposed fields with a scalar detector. The field cross-correlation closely relates to the power spectral density of the superimposed beams. A signal which is equivalent to the TD-OCT A-scan hence can be calculated from the spectrum without mechanically scanning the reference arm, as well. Two different approaches are established to acquire the power spectral density for OCT imaging practically. Spectral domain optical coherence tomography (SD-OCT) systems utilize broadband light sources, which are used for TD-OCT systems as well, and replace the scalar detector of the TD-OCT system with an imaging spectrograph consisting of a spectrometer and a high-speed camera. A single camera frame yields a full spectrum from which the A-scan signal is calculated. High-speed and high-sensitivity cameras are required to enable high A-scan rates, though. In an alternative approach termed swept source optical coherence tomography (SS-OCT), the spectral raw data can be acquired sequentially by using a wavelength scanning laser and a scalar detector such as a photodiode. A single wavelength sweep yields the raw spectral data which is used to calculate the A-scan. SS-OCT systems allow one to use high-sensitivity scalar (point) detectors as well as laser sources which feature a high instantaneous power. High-speed wavelength scanning sources are required to achieve frame rates which are sufficiently fast for real-time OCT imaging. Data processing is similar for SD-OCT and SS-OCT systems. Both techniques calculate the time-domain A-scan signal from the inverse Fourier transform of the spectral raw data and, hence, are described by the more general term Fourier domain optical coherence tomography (FD-OCT). FD-OCT approaches were largely unnoticed, however, until three independent groups demonstrated the technique to yield a superior signal-to-noise (SNR) compared to TD-OCT systems in 2003 [14,15,16]. This discovery triggered a push in OCT development and resulted in most contemporary OCT systems to be based on Fourier domain techniques.

Following the introduction of the basic concepts of OCT, in the remainder, this review paper is organized as follows: important principles of wavefront shaping relevant to application in OCT are described in Section 2, covering the topics adaptive optics (Section 2.1), time reversal and phase conjugation (Section 2.2), iterative wavefront shaping (Section 2.3), and transfer matrix concepts (Section 2.4). Section 3 addresses the implementation of the wavefront shaping principles introduced to current OCT systems. After a short description of typical setups and components developed for this purpose in Section 3.1, Section 3.2 focuses on the exploitation of reflection matrix approaches to suppress multiple-scattered light, whereas Section 3.3 details the spectral and temporal shaping of the scattered light. The application of wavefront shaping techniques for direct OCT signal enhancement and non-invasive focusing is presented in Section 3.4 together with a few representative experimental results. Remaining problems and challenges for future research work are summarized in Section 4 before briefly concluding in Section 5.

## 2. Principles of Wavefront Shaping

Scattering of light is a deterministic process at time scales at which the turbid medium can be considered static. Knowledge of the sample’s scattering properties or of the distortions which are introduced to the optical beam while propagating through the medium, hence, allows for counteracting or even harnessing the effects of scattering for imaging applications. This section explores the principles and fundamentals of wavefront shaping approaches, which enable the focusing of light and imaging in turbid media by manipulating the wavefront of the beam incident on the sample.

### 2.1. Adaptive Optics

Some of the most contemporary devices which exploit active wavefront control for imaging with optically inhomogeneous media are based on adaptive optics. The technique was originally developed to enable diffraction-limited detection and optical focusing in the presence of turbulence and the inhomogeneity of the atmosphere, for example, for astronomic or military purposes [17,18], and can be implemented for microscopic and OCT imaging as well [19,20,21,22,23,24].

**Figure 2:**
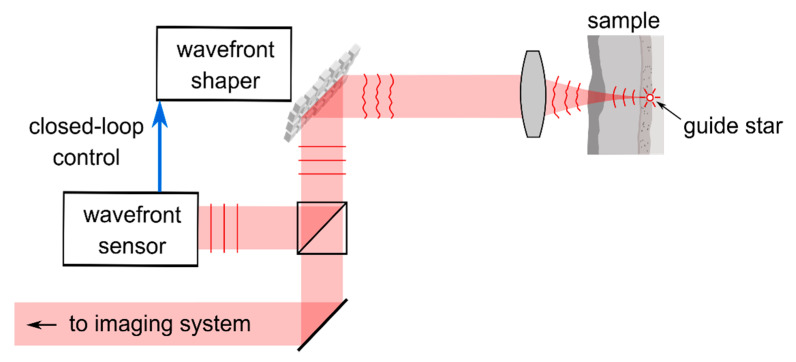
Principle of closed-loop sensor-based adaptive optics. Light emitted from a point-like guide star is detected at a sensor which quantifies aberrations of the wavefront. A closed-loop control is implemented to dynamically correct deviations from the ideal (flat) wavefront with a wavefront shaping element such as a deformable mirror. The approach enables diffraction-limited imaging in the vicinity of the guide star. Image adapted from [19].

A schematic of the general approach is shown in Figure 2. Adaptive optical systems actively control the beam which is backscattered from or incident on the sample under study such that the effects of optical aberrations and scattering are cancelled. Sensor-based approaches require a point-like guide star located near the object which is supposed to be imaged [17]. In case the medium between the object and the imaging system is optically homogeneous, light detected from the guide star can be described by a flat wavefront, i.e., plane of constant phase, in the electromagnetic far field. Inhomogeneity of the medium causes deformations from this ideal wavefront and, in turn, result in a loss of image quality. Hartmann–Shack sensors or holographic techniques are used to detect the shape of the wavefront which is emitted from the guide star after transmission through the turbid medium. A wavefront shaping element such as a deformable mirror (DM), for example, allows one to correct wavefront deformations and, hence, to obtain a diffraction-limited signal from the guide star with the imaging system. The wavefront correction is also valid for light sources located close to the guide star, and hence a diffraction-limited image of the vicinity of the guide star can be obtained as well. It should be noted, however, that multiconjugate adaptive optics is required for more complex and larger imaging areas. Adaptive optics with a single guide star is strictly speaking only valid across the isoplanatic patch. If, however, only defocus is present, moving in and out of the plane is still possible with simple correction.

In another approach, the wavefront correction can be applied to a beam which is incident on the sample to create a diffraction-limited focal spot near the guide star instead. In the context of OCT, high-resolution imaging is thus enabled since the sample can be scanned with a fine focal spot [19,21,23,24]. Methods to create artificial guide stars allow for imaging at an arbitrarily chosen position in the sample. For retinal imaging, for example, a low-NA probe beam for which aberrations at the anterior eye are negligible can be focused on the retina [19].

In strongly scattering samples such as opaque biological tissue, it is not easily possible to create a guide star inside the medium. Some approaches are discussed in Section 2.3.1. In the case that no guide star is available, methods for sensorless wavefront correction can be implemented [20,22]. The techniques try to find an optimal wavefront correction which is applied by the DM and which cancels optical aberrations by optimizing a specific metric of the signal acquired with the imaging system, for example the total image intensity. The approach is similar to iterative wavefront shaping which is discussed in Section 2.3.

A full review of adaptive optical systems is beyond the scope of this article. In general, algorithms used for adaptive optics are tuned for fast wavefront optimization with weakly scattering samples. This is achieved, for example, by estimating the corrective wavefront from a low number of Zernike polynomials [17]. The effect of wavefront correction can be understood by considering the optimized wavefront to counteract aberrations present in the optical system. In contrast, the beam shaping algorithms which are discussed in the remainder are optimized for strongly scattering samples for which adaptive wavefront optimization algorithms are not effective since deformations of the scattered wave are highly heterogeneous, random-like, and may even exceed the spatial resolution of the wavefront shaping element.

### 2.2. Time Reversal and Phase Conjugation

**Figure 3:**
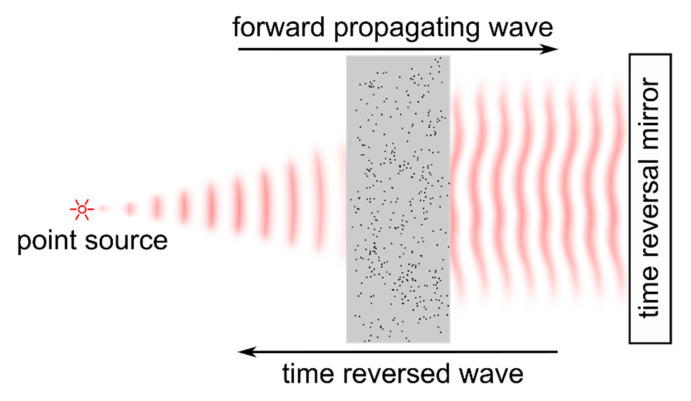
Principle of time reversal and phase conjugation. The field emitted from a (point) source is recorded after propagation through a scattering sample. Applying the time-reversed or the phase-conjugated field to the backside of the sample corresponds to a reversal of propagation direction and recovers the shape of the source field at the front. Image from [8], with permission.

Perhaps the most intuitive way to utilize wavefront shaping approaches to deal with strongly scattering media is based on the time reversal symmetry of the wave equations. Considering the beam which is incident on the turbid sample to be described by its electric field $E_{src}\left(x,y,t\right)$, the field which is detected at a receiver behind the sample is described by the term $E_{out}\left(x,y,t\right)$. In case of monochromatic illumination, a granular (speckle) pattern is observed at the detector. With a pulsed or broadband light source the signal at the receiver is spatially and temporally blurred compared to the incident field. The wave equations which determine the propagation of the electromagnetic field are symmetric with respect to forward and backward travelling waves. Thus, illuminating the sample back-surface with the time-reversed field $E_{out}\left(x,y,-t\right)$ causes the electromagnetic wave to backtrack the propagation in the scattering sample and, thus, to recover the shape of the initial source field $E_{src}\left(x,y,t\right)$ at the sample front face after transmission trough the medium; see Figure 3.

Time reversal experiments were initially demonstrated with acoustic waves which obey wave equations equivalent to those for the electromagnetic field [25]. Ultrasonic transducers enable temporal tracking of the instantaneous pressure wave and can be used both as a source as well as a receiver. Constructing an analog of a *time reversal mirror* from an array of multiple transducers hence allows one to detect the scattered wave spatially and temporally resolved and to directly propagate the time-reversed field back to the medium. In case that a point source in front of the sample is used, the time-reversed acoustic wave which is applied from the other side of the sample creates a spatial focus at the position of the original source and recovers the temporal profile of the source field after propagating through the scattering layer [25,26], Figure 3. The size of the focal spot was shown to correspond to the lateral correlation length of the field which is scattered at the sample. This number can be significantly smaller than the diffraction-limited spot size which is corresponding to the numerical aperture of the time reversal mirror’s transducer array. Further reports demonstrated a time reversal mirror which is consisting of a single transducer element and which, hence, is capable of temporal beam shaping only, to be sufficient for both temporal [26] and lateral [27] focusing of the scattered wave.

**Figure 4:**
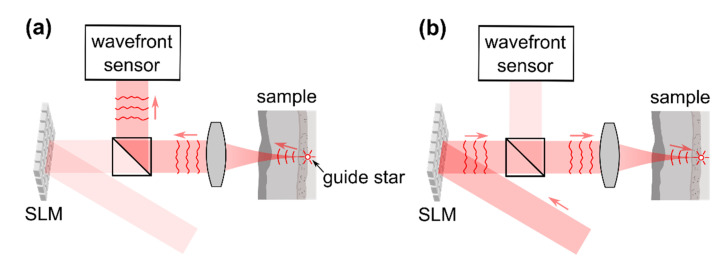
Principle of digital optical phase conjugation. (**a**) Light emitted from a point-like guide star is detected after transmission through the scattering layer. In contrast to adaptive optics, high-resolution wavefront sensing techniques such as phase-shifting interferometry are used. (**b**) A spatial light modulator allows one to propagate the phase-conjugated wavefront back to the sample. A focal spot at the position of the original guide star is created. Image from [8], with permission.

Time-reversal approaches cannot directly be translated to optical radiation since present sensors are not able to temporally track the rapid oscillations of the electromagnetic field. Holographic and interferometric methods allow one to detect and to manipulate the amplitude and phase of monochromatic electromagnetic waves, on the other hand. In case of monochromatic radiation, phase conjugation is equivalent to time reversal. The first application was demonstrated in 1966 by Emmett Leith and Juris Upatnieks who recorded a hologram of an object hidden behind a scattering layer [28]. Utilizing the hologram to apply the phase-conjugated field to the backside of the scattering layer was demonstrated to recover the object’s image at its original position at the other side of the turbid medium. In case the field emitted from a point source, i.e., a focused laser beam or a small fluorescent particle, is holographically detected, the approach allows one to focus light to the position of this guide star by applying the phase-conjugated field to the other side of the turbid layer, as indicated in Figure 3. The technique, hence, is similar to sensor-based adaptive optics which requires a point-like guide star as well, Section 2.1. In 2008, Yaqoob et al. demonstrated phase conjugation with a photorefractive crystal to be feasible for focusing light through thick biological tissue [29]. Purely digital phase conjugation approaches were reported after 2010 and utilize interferometric techniques such as phase-shifting or off-axis interferometry to digitally record the phase of the scattered field and spatial light modulators (SLMs) to create the phase-conjugated beam [30,31,32,33]; see Figure 4. Recent reports demonstrated phase conjugation approaches to be sufficiently fast to focus light through living biological tissue [34,35,36].

#### 2.2.1. Imaging and the Optical Memory Effect

Optical phase conjugation requires a point source or guide star placed behind the scattering layer whose emission can be detected in front of the medium. Applying the phase-conjugated field to the specimen creates a focal spot at the position of the guide star. In principle, this focus can be scanned across the sample for applications such as fluorescence imaging. On the other hand, optical phase conjugation is highly sensitive to minor displacements between the optical system and the scattering sample since the phase-conjugated beam adapts to the sample’s microstructure. In case the sample is moved, the transmitted field decorrelates and the focal spot is lost [29,36]. Judkewitz et al. demonstrated that predominantly forward scattering media allow one to laterally shift the transmitted field without immediately decorrelating by shifting the incident beam (shift/shift correlations) [37]. The effect is valid for small lateral beam displacements only and allows one to scan the focal spot which is created through phase conjugation over a narrow field of view (FOV). This FOV can be sufficient for imaging of microscopic sample structures such as individual cells hidden behind the scattering layer [37,38,39] but, in general, is too small to investigate macroscopic objects.

Hsieh et al. demonstrated scanning of the focal spot behind the scattering layer and imaging by exploiting correlations of the scattered field known as the optical memory effect [31]. In case the field incident on a thin turbid medium is tilted by a small angle, the field which is scattered from the sample is tilted accordingly without fully decorrelating (tilt/tilt correlations) [37,40]. The effect allows one to laterally shift the speckle field which is observed behind the scattering medium or the focal spot created through optical phase conjugation by tilting the beam incident on the sample. The effect is limited to narrow tilt angles and to thin turbid layers, on the other hand. The lateral FOV over which the beam can be effectively scanned before the scattered field decorrelates and the focal spot is lost is proportional to the axial distance between the scattering layer and the focal spot and inversely proportional to the thickness of the turbid medium [37,41]. With biological samples, typically the FOV is limited to a few microns [39].

Imaging, hence, is limited to the close vicinity of guide stars whose emission can be detected to find a phase-conjugated wave. Non-invasive imaging is implemented by embedding virtual guide stars, e.g., fluorescent particles, to the sample [42,43,44]. Such markers may not be distributed homogeneously in the sample, are usually subject to photobleaching and may even be cytotoxic. Xu et al. presented a label-free approach in 2011, termed time-reversed ultrasonically encoded optical focusing (TRUE). The technique uses an ultrasonic transducer to create an acoustic focus in the sample. Light scattered at this focus is frequency shifted due to interaction with the acoustic wave and serves as a virtual point source embedded to the sample. The frequency shifted light is backscattered to the optical system and recorded at a photorefractive crystal which, in turn, allows one to propagate back the phase-conjugated field to the sample and to create an optical focus at the position of the ultrasonic focus. The technique requires single-sided sample access only and was shown to be sufficiently fast to be applied to living biological tissue [45]. The approach was demonstrated with holographic (analogue) [44,45] and with digital phase conjugation systems [46,47]. A focal spot can be created at an arbitrarily chosen target position, depending on the position of the ultrasonic focus. The approach, hence, enables the scanning of macroscopic sample structures for optical imaging.

### 2.3. Iterative Wavefront Shaping

Phase conjugation experiments demonstrate the possibility to create a focal spot from scattered light, provided the correct phase pattern which accounts for scattering at the medium is applied to the beam incident on the sample. For most practical applications, a point-like guide star embedded in the medium and coherent detection of the field emitted from that source are not possible, though. Iterative wavefront shaping approaches, on the other side, enable focusing through scattering media as well, and require to probe the intensity of the scattered field at the position of the supposed focal spot only.

#### 2.3.1. Principles of Iterative Wavefront Shaping

**Figure 5:**
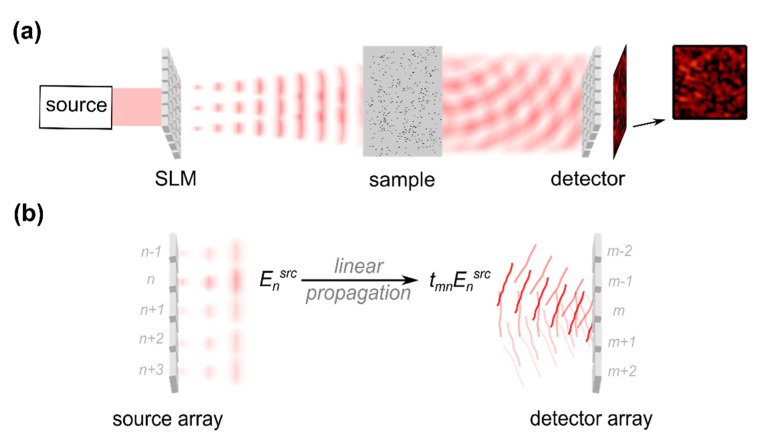
Principle of the optical transmission matrix. (**a**) The beam incident on a scattering sample is modulated by a spatial light modulator (SLM). In case of monochromatic radiation, a speckle pattern is observed behind the sample. (**b**) The field reflected from the SLM is considered to be composed of a number of independent sources indexed n. Since propagation in the sample is linear, the field observed at the m-th detector pixel can be described based on the incident field $E_{n}^{src}$ and the static and complex-valued transmission matrix $t_{mn}$; see Equation (2) [48]. Image from [8], with permission.

In a seminal work, Vellekoop and Mosk first demonstrated focusing through turbid media by iteratively optimizing the shape of the wavefront incident on the sample in 2007 [48]. The approach can be understood by considering the SLM, which is used for wavefront manipulation, to be an array of sources illuminating the sample, see Figure 5a. The phase and amplitude of the individual sources can be controlled electronically, depending on the type of SLM used. In most practical implementations, liquid crystal on silicon (LCOS) devices are employed which enable phase-only wavefront manipulation. Experiments with micro-electro-mechanical systems (MEMS) such as DMs or digital micromirror devices (DMDs) are reported as well and are discussed in Section 2.3.4.

After transmission through the scattering medium, the electromagnetic field features a spatially fluctuating phase and amplitude pattern. The field observed at a detector array behind the scattering medium corresponds to the linear superposition of contributions emitted from the individual source elements, or SLM pixels, as indicated in Figure 5 [48]:

(2) $$E_{m}^{det}=\overset{N}{\underset{n=1}{\sum }}t_{mn}\;E_{n}^{src}$$

The terms $E_{n}^{src}$ and $E_{m}^{det}$ in Equation (2) correspond to the complex field amplitudes at the n-th source and at the m-th detector element, respectively. $t_{mn}$ is the sample’s complex-valued and random-like transmission matrix which describes the linear relation between the incident and the scattered field, compare, e.g., [41].

Considering a single detector pixel with index $m_{t}$, the field observed at this position corresponds to a sum of complex numbers with random amplitude and random phase, see Equation (2). Assuming the individual contributions to be statistically independent and the phase to be uniformly distributed, the amplitude of the scattered field $\left|E_{m_{t}}^{det}\;\right|$ is expected to be Rayleigh distributed, which is the well-known behaviour for monochromatic laser speckle [13,49]. In contrast, in case all contributions $t_{mn}E_{n}^{src}$ from the individual source modes exactly match in phase, the field amplitude as well as the intensity ${\left|E_{m_{t}}^{det}\right|}^{2}$ become maximal. A high intensity, i.e., a focal spot, results at the target pixel $m_{t}$, which can be understood to be an effect of constructive interference of the scattered field.

**Figure 6:**
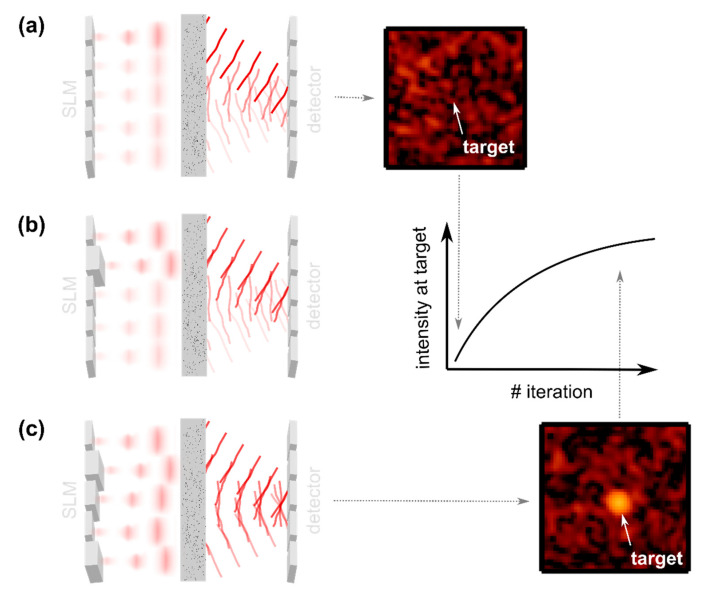
Principle of iterative wavefront shaping. (**a**) A flat wavefront incident on a scattering sample results in a speckle pattern to be observed at a detector placed behind the medium. (**b**) The phase of individual segments of the incident wavefront can be manipulated using an SLM. Due to the linearity of propagation, the phase of the respective contributions to the scattered field shifts accordingly. A high intensity at an arbitrarily chosen target at the detector is observed if a high number of field contributions match in phase at this position. The wavefront shaping algorithm iteratively optimizes the phase pattern applied to the incident beam such that the intensity at the target is maximized. (**c**) A high-intensity focal spot on top of a speckle pattern results with the final optimized wavefront. Image from [8], with permission.

The sample’s transmission matrix $t_{mn}$ is static, but the phase of the incident field $E_{n}^{src}$ can experimentally be manipulated with the SLM. Note that this is only true for a static sample. A biological sample would degrade the matrix quickly; also see Section 2.3.4. The optimized phase pattern which is applied by the SLM is found by probing the intensity at the target position $m_{t}$ at which the focus is supposed to be created and by iteratively optimizing the phase pattern such that the intensity is maximized. The approach is illustrated in Figure 6. Algorithms for wavefront optimization are discussed in Section 2.3.3.

The most straight forward way to probe the intensity of the scattered field is to place a detector behind the turbid medium. Detector-based approaches allow one to investigate the impact of wavefront shaping on the scattered field and to test dependencies on experimental parameters. The lateral size of the shaped focus is found to correspond to the lateral correlation length, i.e., the speckle size, of the scattered field [50], similar to phase conjugation approaches [25,26]. The focal spot is observed to be created on top of a speckled background, Figure 6. The local intensity enhancement at the target compared to the out-of-target intensity rises with an increasing number N of source elements, which corresponds to the number of independent wavefront segments controlled by the SLM [48,51,52], and drops with increasing target size [51,52,53]. A number of theoretical investigations found a linear dependence on the number of wavefront segments in case the transmission matrix is assumed to obey Gaussian scattering statistics [48,50,53,54,55]. The efficiency of iterative wavefront shaping further depends on the type of wavefront modulation used. Naturally, the approach is expected to perform best in case the phase and the amplitude of the incident beam can be manipulated [50,55]. Most practical implementations are based on phase-only wavefront control enabled with LCOS SLMs. The peak intensity of the focal spot is expected to read approximately 78% of the value achieved with full-complex wavefront control [48,50,53,55]. Binary amplitude-only wavefront control, which is for example realized with digital micromirror devices (DMDs), results in an expected focal spot intensity of approximately 16% of the value achieved with complex-valued wavefront control [54].

#### 2.3.2. Feedback Types and Implementation for Imaging

Imaging based on iterative wavefront shaping is, similar to optical phase conjugation, Section 2.2.1, performed either by creating a single focal spot inside the sample and scanning this focus using the optical memory effect or by sequentially scanning the position at which the focus is created. The former method is limited to a narrow FOV at which a single optimized wavefront is able to create a focus behind the turbid layer before the scattered field decorrelates. The latter technique requires to optimize the wavefront which is incident on the sample at each lateral scan position anew. Opposed to optical phase conjugation, which is a single-shot technique, iterative wavefront shaping typically requires a high number of acquisitions to find a single optimized wavefront. The latter approach, thus, faces serious challenges regarding the acquisition speed.

Iterative wavefront shaping techniques require some means to determine the intensity of the scattered field at the target at which a focal spot is supposed to be created only, but no guide star whose complex-valued field is detected after transmission through the scattering layer; compare Section 2.2. Vellekoop et al. demonstrated non-invasive focusing by embedding fluorescent particles to the sample which can be used as point-like intensity probes [53]. Wavefront shaping is enabled by maximizing the total fluorescence emission which is detected in front of the sample. In case fluorescence from multiple particles is detected simultaneously, the observed signal does not reflect the intensity at a single spatially confined target, on the other hand, and the wavefront shaping algorithm can fail to create a single focal spot. To overcome this problem a non-linear probe, for example, the two-photon fluorescence signal emitted from a small particle, can be taken to create a feedback for the wavefront shaping algorithm instead [56,57]. Indeed, Katz et al. showed a non-linear optical feedback to be necessary to focus light in case the fluorophores are densely packed [58]. Once the optimized wavefront is found, the focal spot can be scanned for fluorescence imaging behind the scattering layer [38,39,56,58]. The FOV of the technique is limited to the vicinity of the intensity probe which was the original target for focusing (compare Section 2.2.1).

Non-invasive and label-free wavefront shaping was demonstrated similar to TRUE (Section 2.2.1) with feedback probes created from light which is scattered at a focused acoustic wave [59] or by measuring the amount of light which is locally absorbed at the target position. The local absorption can be quantified from the sample’s photoacoustic response [60,61,62,63], which is the acoustic signal detected with an ultrasonic transducer after absorption of a short laser pulse in the sample. Both techniques probe the intensity at the focal position of the transducer and, thus, allow one to scan the position at which the wavefront shaping algorithm creates an optical focus by scanning the focus of the transducer [62,64]. The combination of wavefront shaping and photoacoustic imaging is of particular interest since the width of the optical focus can be significantly smaller than the width of the transducer’s diffraction-limited acoustic focus [60,61]. Detection of the acoustic signal after sample excitation with a focused beam, thus, enables photoacoustic imaging below the resolution limit of the transducer [61]. Tzang et al. proposed an alternative method to quantify the local absorption at the target which is based on the direct detection of the sample’s thermal expansion with an OCT system [65]. The approach is complementary to photoacoustic feedback and enables non-invasive optical focusing, as well. In contrast to photoacoustic feedback, the technique cannot directly be applied for imaging, on the other hand, and the penetration depth is limited by the penetration depth of the OCT system, which, for most biological samples, is low compared to photoacoustic systems. Non-invasive wavefront shaping directly based on the OCT signal was first demonstrated in 2012 and is discussed in detail in Section 3.

#### 2.3.3. Algorithms

Here, we provide a brief discussion on some of the most popular algorithms for iterative wavefront shaping which were implemented. In general, the algorithms try to find an optimized pattern to be applied to the SLM such that the feedback signal, which reflects the intensity at the target, is maximized, as indicated in Figure 6. In most practical implementations the modulated beam is either imaged or focused from the plane of the SLM to the sample. Due to their iterative nature, wavefront shaping algorithms require no knowledge of the sample’s optical properties and correct for aberrations and misalignments present in the optical setup as well.

The number of degrees of freedom of the wavefront incident on the sample matches the pixel count of the SLM, which typically is in the range of $10^{6}$ or higher for most modern devices. This number is too high to optimize iteratively within a reasonable time span and, thus, most algorithms group multiple pixels to larger segments with uniform amplitude and phase each. The number of degrees of freedom of the incident wavefront matches the number of independent segments in this case.

The algorithm originally proposed by Vellekoop and Mosk optimizes the phase delay at each individual wavefront segment one after another [48,66]. At each segment, a number of different phase delays is tested (e.g., ten uniformly sampled phases from 0 to 2 π) and the phase which results in a maximum intensity at the target is chosen, respectively. The full wavefront is constructed by sequentially repeating this procedure for all segments within the beam aperture. The run-time of the algorithm, thus, depends on the number N of optimized wavefront segments and on the number m of different phase-values applied to each segment. Full wavefront optimization, thus, requires one to apply mN phase patterns to the SLM and to measure the resulting intensity at the target position, respectively.

The sequential algorithm probes the field which is reflected from a single SLM segment one at a time. In case a high number of segments, i.e., small segment sizes, are used, the algorithm becomes sensitive to experimental noise due to the low intensity of light reflected from the individual segments. To overcome this problem, phase patterns which span the full aperture of the beam at once are tested instead. The approach was first demonstrated with the partitioning algorithm presented by Vellekoop and Mosk in 2008 [66]. Conkey et al. presented a genetic algorithm in 2012 which has been used in a high number of wavefront shaping experiments reported in literature since; also see [64]. The genetic algorithm creates an initial set, or population, of random phase patterns. Each phase pattern is ranked according to the intensity which results at the target once it is applied to the SLM. The patterns are optimized by repeatedly creating new generations of phase masks from the previous population. New phase patterns are blended from two randomly chosen phase masks from the previous generation, whereas the selection probability rises with increasing rank. Additionally, random fluctuations are included to newly generated patterns (mutation). The mutation probability is set to drop for later generations to allow the algorithm to converge. The genetic algorithm was demonstrated to outperform the sequential algorithm in the presence of experimental noise and with temporally unstable samples [64].

#### 2.3.4. Acquisition Time

Acquisition time is a critical factor when the technique is supposed to be applied to living biological samples. Due to macroscopic sample movements, respiration, blood flow and cellular movement, the physical structure of the sample quickly changes and the scattered field decorrelates. As a consequence, the focal spot created through optical phase conjugation or through iterative wavefront shaping quickly decays at time scales down to a few milliseconds [34,35,36,45].

The acquisition time required to find an optimized wavefront which focuses scattered light to a single spot depends, in general, on the number N of independent segments or degrees of freedom of the incident wavefront, on the number m of signal acquisitions which are required to find the optimal phase for each wavefront segment, and on the time required for each individual signal acquisition. The efficiency of wavefront shaping is expected to rise with increasing number N which, hence, is typically chosen to be as high as possible within a reasonable optimization time; see Section 2.3.1. The number of acquisitions m which are required to find the optimal phase for each wavefront segment is determined by the optimization algorithm. If the modulation characteristics of the SLM is unknown, this number needs to be high in order to find the phase iteratively. With a well-defined modulation characteristics techniques similar to phase-shifting interferometry can be employed to find the optimal segment phases. A minimal number of m=3 acquisitions for each wavefront segment results. The technique is similar to transmission matrix approaches which are discussed in Section 2.4. A number of high-speed wavefront shaping systems are reported which are based on a parallelized optimization algorithm originally reported by Cui in 2011 [67]. The algorithm requires, in principle, a minimum number of m=2 acquisitions per degree of freedom of the incident wavefront. Choi et al. demonstrated another optimization algorithm in 2013 which enables wavefront shaping with spectral domain OCT (SD-OCT) systems and which requires a single (m=1) acquisition for each segment of the optimized wavefront [68]. The technique is strongly related to transmission matrix approaches presented in Section 2.4.

The time required for a single signal acquisition depends on the speeds of wavefront control and feedback detection. Typically, the acquisition speed is limited by the frame rate of the spatial light modulator rather than by the detector. Liquid crystal devices which are often used for phase-only wavefront manipulation feature response times in the range of several milliseconds and, thus, are not suited for fast systems. Most high-speed wavefront shaping approaches, thus, are based on micro-electro-mechanical systems such as DMs [38,39,56,69,70] or DMDs [71,72] which feature frame rates in the kHz up to the MHz range and which, hence, enable wavefront optimization well below 1 ms per independent wavefront segment [69,72]. DMs enable phase-only modulation but come with a low number of independent pixels. DMDs, on the other hand, feature a high pixel count but enable binary amplitude (on/off) modulation only, which results in a reduced efficiency of the optimized wavefront compared to phase-only wavefront control, Section 2.3.1. Recently, Feldkhun et al. implemented Cui’s parallel wavefront shaping algorithm [67] with wavefront manipulation based on acousto-optic modulators [73]. The approach was shown to be extremely fast and enabled wavefront shaping with N=100 independent wavefront segments in only 10 μs.

### 2.4. Transmission Matrix Approaches

Optical propagation in a scattering but static sample is considered to be a linear and time-invariant process in case the intensity of the electromagnetic field is low. The linear relation between the scattered field and the field incident ono the sample can be described through the complex-valued transmission matrix $t_{mn}$ which quantifies the sample’s scattering properties (Equation (2)). Knowledge of the transmission matrix allows one to reconstruct the incident field by detecting the scattered field, i.e., for imaging, or to optimize the wavefront incident on the medium such that an arbitrary field distribution is created after scattering, for example for focusing similar to iterative wavefront shaping.

#### 2.4.1. Principles of Transmission Matrix-Based Concepts

**Figure 7:**
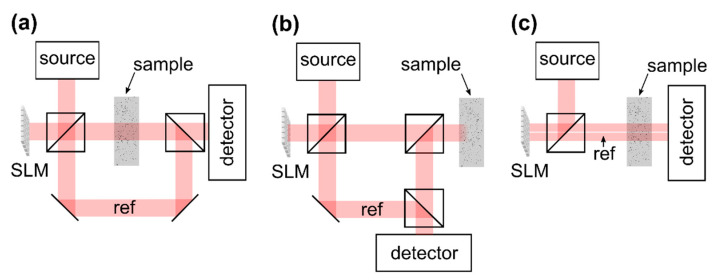
Experimental designs for transmission matrix acquisition. (**a**) Transmission geometry with external reference beam. (**b**) Reflection geometry with reference beam. (**c**) Self-referenced transmission geometry. Abbreviations: SLM—spatial light modulator, ref—reference beam. Image from [8], with permission.

In principle, the transmission matrix can easily be determined experimentally according to the definition given in Equation (2). Switching on of a single segment of the beam incident on the sample ($E_{n}^{src}=1$ if $n=n^{\prime }$, zero otherwise) and detecting the complex-valued field $E_{m}^{det}$ which is scattered to a detector placed behind the medium directly yields the column of the reflection matrix $E_{m}^{det}=\overset{N}{\underset{n=1}{\sum }}t_{mn}E_{n}^{src}=t_{mn^{\prime }}$ which corresponds to the respective wavefront segment. The full matrix is determined by iterating all segments. This approach is kept for almost all practical transmission matrix acquisition methods which are reported in the literature. The measurement of the complex-valued scattered field necessitates interferometric acquisition techniques which, in turn, require one to superimpose the scattered field with a static reference beam, Figure 7a,b. In many practical applications, implementation of a reference beam which is bypassing the sample is not possible, however, since an optical access to the sample back surface is not available or not practical.

**Figure 8:**
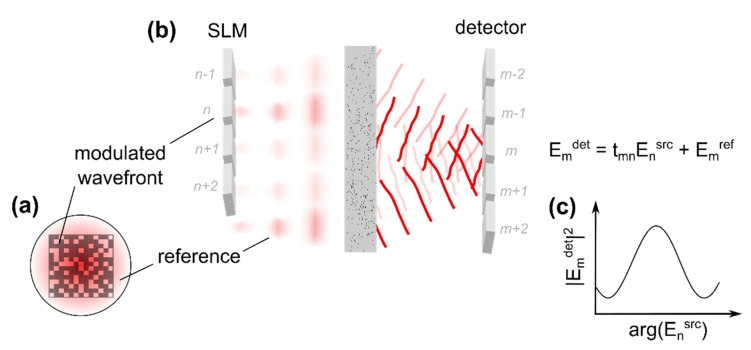
Self-referenced transmission matrix measurement. (**a**) Beam in the plane of the SLM. A fraction of the beam incident on the sample is modulated. Another part of the beam remains static and serves as a reference for interferometric acquisition. (**b**) The modulated and the static reference beam are both scattered at the sample and interfere at the detector (compare Figure 5). (**c**) A sinusoidal intensity fluctuation is observed in case the phase of the modulated part of the wavefront is changed. The phase and amplitude of the scattered field which results from sample illumination with the modulated wavefront segment, i.e., one column of the transmission matrix, can be determined from this signal using phase-shifting algorithms. Image from [8], with permission.

In 2008, it was reported by Vellekoop et al. that during iterative wavefront shaping a sinusoidal intensity fluctuation is observed at the target if a single wavefront segment is modulated while the rest of the beam remains static [53]. A brief analysis of this observation reveals that a fraction of the beam which is reflected at the SLM without being modulated can be used as reference for the interferometric acquisition as well, even though this beam is scattered at the sample, Figure 7c and Figure 8a. The approach allows one to determine the transmission matrix without additional external reference beam.

Similar to the previous section, the electric field incident on the sample is assumed to read $E_{n}^{src}$ in the plane of the spatial light modulator, where n is the index of the respective wavefront segments, Figure 5. Considering modulation at the $n^{\prime }$-th segment only while leaving the rest of the beam static, the field which is scattered to the m-th pixel of a detector placed behind the medium reads (Figure 8b):

(3) $$E_{m}^{det}=t_{mn^{\prime }}\;E_{n^{\prime }}^{src}+E_{m}^{ref}$$

The term $E_{m}^{ref}$ corresponds to those parts of the beam which are not manipulated by the SLM and which are scattered to the m-th element of the detector, as well. This term, hence, is considered static and reads $E_{m}^{ref}=\overset{N}{\underset{n=1\neq n^{\prime }}{\sum }}t_{mn}E_{n}^{src}$ according to Equation (2). The intensity at the detector in case only the phase $\phi_{n^{\prime }}=\arg\left(E_{n^{\prime }}^{src}\right)$ of the modulated wavefront segment is changed is found from the interference law [53]:

(4) $$I_{m}^{det}\left(\phi_{n^{\prime }}^{src}\right)\propto {\left|t_{mn^{\prime }}\right|}^{2}\;{\left|E_{n^{\prime }}^{src}\right|}^{2}+\;{\left|E_{m}^{ref}\right|}^{2}+2\;ℜ\left\{\Gamma_{m}\left(\phi_{n^{\prime }}^{src}\right)\right\}$$

The interference term (third term) corresponds to the real part of the field cross-correlation $\Gamma_{m}$ [53]:

(5) $$\Gamma_{m}\left(\phi_{n^{\prime }}^{src}\right)=t_{mn^{\prime }}\;{\left(E_{m}^{ref}\right)}^{*}\;E_{n^{\prime }}^{src}\;=\left|t_{mn}\right|\;\left|E_{m}^{ref}\right|\;e^{i\arg\left(t_{mn^{\prime }}\right)-\phi_{m}^{ref}}\;\left|E_{n^{\prime }}^{src}\right|\;e^{i\;\phi_{n^{\prime }}^{src}}$$

Obviously, one finds $ℜ\left\{\Gamma_{m}\right\}\propto \cos\left(\phi_{n^{\prime }}^{src}\right)+const.$, i.e., the intensity at the detector features a cosine fluctuation in case the phase $\phi_{n^{\prime }}^{src}$ of the $n^{\prime }$-th wavefront segment is modulated, Figure 7c.

In principle, the term $\Gamma_{m}$ probes the amplitude and the phase of the transmission matrix $t_{mn}$. This information can be used to find an optimized wavefront which results in focusing at the detector, similar to iterative wavefront shaping [53]. A major generalization of the concept was proposed by Popoff et al. who presented the first experimental acquisition of the optical transmission matrix in 2010 [74].

First, instead as with a single point detector as presented by Vellekoop et al. [53], the scattered field can be investigated at a large detector array with a high number of pixels simultaneously. This consideration is included to the previous equations through the subscript m, which reflects the index of the detector element or detector pixel.

Second, Popoff et al. found that the transmission matrix can be recovered from the interference signal through algorithms well-known from phase-shifting interferometry. The group proposed a four step algorithm which reconstructs the transmission matrix from the intensity $I_{m}^{det}\left(\phi_{n^{\prime }}^{src}\right)$ (Equation (3)) which is detected with four discrete phase shifts $\phi_{n^{\prime }}^{src}$ applied to the modulated part of the incident beam, respectively [55,74]. Taking Equations (4) and (5) into account, this algorithm yields:

(6) $$t_{mn^{\prime }}^{obs}=\frac{1}{4}\left[\left(I_{m}^{det}\left(\phi_{n^{\prime }}^{src}=0\right)-I_{m}^{det}\left(\pi \right)\right)+i\;\left(I_{m}^{det}\left(\frac{3\pi }{2}\right)-I_{m}^{det}\left(\frac{\pi }{2}\right)\right)\right]\;=t_{mn^{\prime }}\;{\left(E_{m}^{ref}\right)}^{*}$$

This is the $n^{\prime }$-th row of the observed transmission matrix $t_{mn}^{obs}$. The exact transmission matrix $t_{mn}$ is not accessible experimentally since the reference field $E_{m}^{ref}$ which is used for the interferometric acquisition results from parts of the beam which are scattered at the sample and, hence, have an unknown amplitude and phase profile (Figure 8).

Third, in a more general way, the transmission matrix $t_{mn}$ describes the linear relation between the complex amplitudes $E_{n}^{src}$ of the n-th optical basis mode incident on the sample and the amplitude $E_{m}^{det}$ at the m-th scattered mode [55,74]. The modes correspond to an (arbitrarily chosen) orthogonal basis of the electromagnetic field at the planes of wavefront manipulation and detection, respectively. So far, we considered wavefront manipulation on a pixel-by-pixel basis in both planes only. This basis is indeed shown to be orthogonal [75] and is well suited to describe the scattered field in the plane of the detector since it aligns well with the pixelated signal received with a scientific camera, for example.

In case a pixel-by-pixel or segment-by-segment basis is chosen to describe the field which is incident on the sample, similar to iterative wavefront shaping the system can be sensitive to experimental noise (compare Section 2.3.3). The individual basis modes correspond to spatially non-overlapping segments of the wavefront and, thus, their respective intensity is low compared to the total power of the beam. Instead, the transmission matrix can be determined for a set of modes which span a large fraction of the beam. Typically, a plane wave or a Hadamard basis is chosen. The individual modes of the plane wave basis can be created with a phase-only SLM by applying a set of linear phase ramps with a different tilt angle for each mode, respectively [68]. In case of a Hadamard basis, the phase patterns which create the individual modes correspond to the respective rows of a Hadamard matrix [55,74]. The values of the Hadamard matrix read 1 or −1, which correspond to phase delays of 0 and π. The row-vectors of the matrix are mutually orthogonal by definition. The total number of modes matches the dimension of the Hadamard matrix and typically is a power of two since the matrix can easily be constructed for this case.

To summarize, the optical transmission matrix is acquired experimentally by using an SLM to sequentially apply a set of basis modes to the beam which is incident on the sample. Each mode spans a large fraction of the beam, but a part of the beam remains unmodulated to provide a static reference for the interferometric acquisition, Figure 8a. A detector which is placed behind the scattering sample captures the intensity of the scattered field Figure 8b. The SLM is used to shift the phase offset of the respective mode which is incident on the sample, but not the phase of the static part of the wavefront. Multiple acquisitions with different phase delays allow one to reconstruct the complex-valued scattered field from the intensity which is captured at the detector, Equations (4) and (6). The field which results from sample illumination with a single mode corresponds to a single column of the observed transmission matrix Equation (6). The full matrix is captured by repeating this procedure for all modes.

#### 2.4.2. Single-Point Focusing

Once the transmission matrix is experimentally determined, a wavefront which creates a focal spot from scattered light at the $m_{t}$-th detector pixel, similar to iterative wavefront shaping, can be calculated from the complex-conjugate of the corresponding matrix row [53,55,74]:

(7) $$E_{n}^{src,opt}={\left(t_{m_{t}n}^{obs}\right)}^{*}$$

The phase of the incident wavefront reads $\arg\left(E_{n}^{src,opt}\right)=\arg\left(t_{m_{t}n}\right)+\phi_{m_{t}}^{ref}$ according to the definition of the observed transmission matrix (Equation (6)). The intensity at the detector once the optimized wavefront is applied to the sample is evident by inserting Equation (7) in Equation (2):

(8) $$I_{m}^{det}\propto {\left|\overset{N}{\underset{n=1}{\sum }}\left|t_{mn}\right|\;e^{i\arg\left(t_{mn}\right)}\;\left|E_{n}^{src}\right|\;e^{\;i\left(-\arg\left(t_{m_{t}n}\right)+\phi_{m_{t}}^{ref}\right)}\right|}^{2}$$

For an arbitrarily chosen detector element (m≠m_t), Equation (8) corresponds to a sum of random phasors. In contrast, at the target pixel $m_{t}$ the individual contributions add up in phase and, indeed, a high intensity results:

(9) $$I_{m_{t}}^{det}\propto {\left|\overset{N}{\underset{n=1}{\sum }}\left|t_{m_{t}n}\right|\;\left|E_{n}^{src}\right|\right|}^{2}{\left|e^{i\;\phi_{m_{t}}^{ref}}\right|}^{2}$$

Utilizing the transmission matrix for single-point focusing is equivalent to iterative wavefront shaping [55] and, hence, the same considerations regarding the focusing efficiency apply; see Section 2.3.1. In contrast to the previous approach, the transmission matrix needs to be determined once and the optimized wavefront can directly be calculated afterwards without further acquisitions or iterative optimization algorithms. The transmission matrix describes the scattered field at the complete FOV of the detector, and, hence, a wavefront which creates a focal spot from scattered light at any position within the FOV can be found without needing to reacquire the matrix. Furthermore, multiple wavefronts optimized for different target positions can be superimposed for simultaneous focusing [74]. To this end an m-element target vector $E_{m}^{det,target}$, which describes the supposed field at the detector after wavefront shaping, may be defined. The vector elements, for example, are chosen to be unity if the index m corresponds to a detector pixel at which a focus is supposed to be created and zero otherwise. The optimized incident wavefront reads in this case according to Equation (7) [74]:

(10) $$E_{n}^{opt}=\;\overset{M}{\underset{m=1}{\sum }}{\left(t_{mn}^{obs}\right)}^{*}\;E_{m}^{det,target}$$

#### 2.4.3. Imaging Using Transmission Matrix Approach

Transmission matrix as well as iterative wavefront shaping approaches both enable focusing of scattered light but require some means to determine the intensity of the scattered field after transmission through the medium. In contrast to iterative wavefront shaping, which tries to maximize the intensity at the target by iteratively optimizing the incident wavefront, see Figure 6, transmission matrix approaches track small intensity fluctuations which result from interference of the phase-modulated wavefront with a static reference field, as indicated in Figure 8. A number of iterative wavefront shaping algorithms utilize this effect as well, and cannot clearly be distinguished from transmission matrix approaches, for example, the parallelized algorithm which was presented by Cui [67] and implemented with a number of high-speed wavefront shaping systems (Section 2.3.4). As a major difference, the transmission matrix determines the scattered field at a spatially extended FOV simultaneously, in contrast to point detection in case of iterative wavefront shaping. Transmission matrix approaches, thus, enable focusing at any point within the FOV once the matrix is determined whereas iterative wavefront shaping approaches require to repeat the optimization algorithm at each target position.

Non-invasive techniques to determine the local optical intensity inside a scattering sample were demonstrated for iterative wavefront shaping, Section 2.3.2, and can be applied to transmission matrix approaches, as well. Chaigne et al., for example, demonstrated the acquisition of the transmission matrix from the photoacoustic response of a scattering sample [76]. Choi et al. demonstrated the acquisition of the matrix and optical focusing based on the sample’s OCT signal [68]. This approach is discussed in detail in Section 4. Imaging can be performed by scanning the position of the focal spot, similar to the methods discussed in Section 2.3.2. In contrast to the iterative wavefront shaping, utilizing the transmission matrix enables scanning beyond the correlation length of scattered light since the matrix can be used to recalculate new wavefronts for focusing at different scan positions. The FOV of the approach is limited to the FOV of the detector which was used to determine the transmission matrix.

Additionally, knowledge of the transmission matrix in principle allows one to directly reconstruct the electromagnetic field which is emitted from a hidden object by detecting the scattered field after transmission through the turbid layer [55,74,77,78]. The technique enables imaging through the scattering layer but requires detailed knowledge of the transmission between the object and the detection plane, i.e., calibration of the imaging system with a high-resolution wavefront measurement in the plane of the hidden object. The approach was demonstrated for imaging with a static scattering layer placed in a microscopic setup [77] but is not feasible for imaging with dynamically changing (living) biological tissue.

Similar to the transmission matrix, the reflection matrix of a scattering sample can be determined by changing the detection geometry [79,80]. The approach requires single-sided sample access only and, thus, allows one to utilize a static reference beam for interferometric detection of the back-scattered light (Figure 7b). As a consequence, fast single-shot interferometric acquisition techniques such as off-axis holography can be used [79]. Knowledge of the reflection matrix allows one to investigate the sample’s scattering properties and enables the focusing of back-scattered light similar to the transmission matrix in Section 2.4.2. The application to imaging is not as straight-forward as with the transmission matrix, on the other hand, since light backscattered from different depths of the sample is detected simultaneously. A number of groups utilized time-of-flight gating to detect the reflection matrix with light backscattered from a selected depth only. The technique combines reflection matrix approaches with OCT imaging and is discussed in Section 3.2 in detail.

#### 2.4.4. Singular Value Decomposition

A singular value decomposition of the transmission or reflection matrix allows one to quantify the scattering properties of the sample. A detailed discussion of this effect is beyond the scope of article, however. Recent studies further demonstrated correlations between modes which correspond to large eigenvalues and strongly scattering particles which are embedded in the sample. These correlations enable non-invasive focusing and imaging.

The DORT method (French acronym for decomposition of the time reversal operators) tries to identify dominant scatterers present in the turbid sample from a singular value decomposition of the time reversal operator $TT^{†}$ [81]. This operator is calculated from the reflection or from the transmission matrix T and, in principle, describes the relation between the target field distribution for optical phase conjugation and the resulting scattered field after application of the phase-conjugated wave to the sample (compare Equations (2) and (10)). The eigenvalues of the time reversal operator yield modes which are scaled by the phase conjugation process only, i.e., modes for which the scattered field after phase conjugation actually matches the target light distribution. The eigenvalues quantify the corresponding scaling factors. In many practical implementations the singular value decomposition of the time reversal operator $TT^{†}$ is found from the decomposition of the transmission matrix T instead, which yields equivalent eigenvectors [80].

Prada et al. demonstrated in the context of ultrasonic time reversal (Section 2.2) that large eigenvalues of the time reversal operator are associated with dominant scatterers present in the sample [81]. Namely, in case single-scattered waves are detected only, a one-to-one association exists and, hence, coupling the wavefront to the sample which corresponds to the largest eigenvalue creates a focus at the strongest reflecting particle. Popoff et al. implemented the approach with the optical reflection matrix acquired from a scattering sample and demonstrated non-invasive focusing to strongly reflecting particles hidden inside the medium [80]. Imaging in the vicinity of dominant scatterers can be performed similarly to iterative wavefront shaping by exploiting the optical memory effect to laterally scan the focal spot (Section 2.3.2). In another approach, an image of dominant scattering particles embedded to the sample can directly be constructed from the eigenvectors corresponding to the largest eigenvalues of the time reversal operator as well [82].

The approach is valid in case the detected reflection matrix is dominated by single-scattered waves only, however [80,81]. The application to strongly scattering samples was recently demonstrated by Badon and is based on the suppression of signal contributions from multiple-scattered light prior to the singular value decomposition of the matrix [82]. The approach is similar to full-field OCT and is discussed in Section 3.2. Jeong et al. demonstrated the singular value decomposition with a comparable system to enable non-invasive focusing in the presence of multiple-scattered light, see Section 3.4.2.

#### 2.4.5. Acquisition Speed Enhancement

The considerations relevant for iterative wavefront shaping (Section 2.3.4) are also valid for the acquisition speed of transmission and reflection matrix approaches: the total acquisition time depends on the number of independent modes or wavefront segments incident on the sample, on the number of signal acquisitions per mode (three for phase-shifting algorithms, one for off-axis holography), and on the speed of the detector and the SLM. High-speed systems, as required by many in vivo applications, among others, demand for fast SLMs for wavefront manipulation. In principle, most approaches discussed in Section 2.3.4 can be employed for this purpose as well.

## 3. Applications of Wavefront Shaping to Optical Coherence Tomography

Optical coherence tomography utilizes confocal and coherence gating to suppress multiple-scattered light and to enable imaging in turbid media. In practical applications, the rejection of multiple-scattered light is not complete, however, and a significant fraction of the OCT signal arises from multiple small-angle scattering events. These signal contributions reflect the sample morphology, as well, and become relevant when strongly forward scattering media such as biological tissue are imaged.

This section discusses current state-of-the-art techniques to combine OCT and wavefront shaping for depth-enhanced imaging with scattering samples. In principle, two approaches are reported to date. The acquisition of the sample’s reflection matrix combined with full-field OCT enables the additional rejection of multiple-scattered light for depth-enhanced imaging in turbid samples. The technique exploits correlations between the incident beam and the single-scattered reflected wave and is discussed in Section 3.2. In another approach, iterative wavefront shaping or optical phase conjugation based on the reflection matrix is used for the non-invasive focusing of multiple-scattered light inside the sample. The OCT signal depends linearly on the amplitude of the electromagnetic field and, thus, the received signal can directly be enhanced by focusing small-angle scattered light to the detection volume. This technique can be implemented with full field as well as with scanning OCT systems. A detailed discussion of the approach is given in Section 3.3 and Section 3.4. Remaining challenges to be solved in the future are briefly described in Section 4.

### 3.1. OCT Implementations

**Figure 9:**
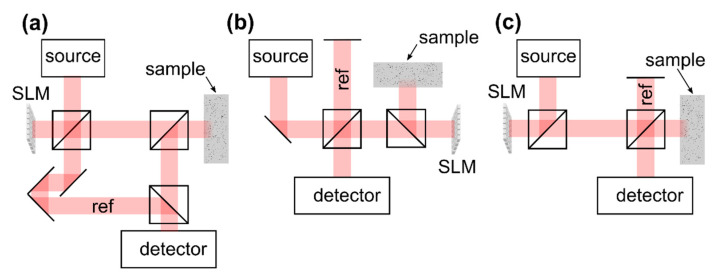
Optical coherence tomography (OCT) designs enabling wavefront manipulation. (**a**) Mach–Zehnder design. The SLM manipulates the sample beam only [68,82,83,84,85,86]. (**b**) SLM at sample arm. The sample beam passes the SLM again after reflection at the sample [70]. (**c**) SLM at source beam. The sample and the reference beam are affected simultaneously by wavefront manipulation with the SLM [71,87,88,89]. Abbreviations: *SLM*—spatial light modulator, *ref*—reference beam. Image from [8], with permission.

The reflection matrix as well as iterative wavefront shaping techniques require to include an SLM to the OCT system which enables wavefront manipulation at the beam illuminating the sample. Designs which are reported to date are illustrated in Figure 9, assuming a reflective SLM is used. The Mach–Zehnder design (Figure 9a) allows one to separate the reference from the sample beam and enables independent wavefront shaping by including the SLM to the sample beam only. The field which is backscattered from the sample is not manipulated again at the SLM prior to detection. On the other hand, the design is rather bulky, requires a high number of optical components and a high mechanical stability, and cannot directly be implemented to existing OCT systems which are based on a Michelson interferometer. Figure 9b illustrates a Michelson interferometer-based OCT design. Placing the SLM at the sample beam enables wavefront shaping while leaving the reference beam static. The field which is backscattered from the sample passes the SLM again prior to detection, on the other hand. Figure 9c illustrates another design based on a Michelson interferometer. Placing the SLM at the source beam allows one to feed the shaped wavefront to a conventional OCT scan head which includes the optics of the reference and the sample beam [71]. The approach causes the shaped wavefront to be coupled to the reference beam as well. As a consequence, the reference beam does not remain static during the acquisition of OCT signals with differently shaped wavefronts applied.

### 3.2. Exploiting the Reflection Matrix to Suppress Multiple-scattered Light

The reflection matrix describes the linear dependence between the backscattered field and the field which is incident on the sample. Typically, light reflected from different depths is detected simultaneously and, hence, the matrix cannot directly be used for imaging; compare Section 2.4.3. The acquisition of the reflection matrix requires single-sided sample access only and, in contrast to transmission matrix approaches, a static reference beam can be added for interferometric acquisition of the complex-valued backscattered field, as illustrated in Figure 7. Utilizing a broadband instead of a monochromatic light source allows one to detect the reflection matrix time-of-flight or depth-selectively. The approach is similar to time domain OCT (TD-OCT) since interference at the detector is only observed from the fraction of backscattered light whose optical path length matches the length of the reference beam. The reference arm length, hence, determines the depth from which backscattered light is detected during the acquisition of the matrix.

Experimental devices to capture the time-gated reflection matrix are similar to full-field OCT (FF-OCT) systems with wavefront manipulation at the sample beam. In contrast to conventional FF-OCT systems, a spatially coherent source needs to be employed to enable beam shaping with the SLM. The reference beam is supposed to remain static and the field which is reflected from the sample should not pass the SLM again between scattering and detection. Hence, all works demonstrating the experimental acquisition of the time-gated reflection matrix are based on the Mach–Zehnder design to date, Figure 9a [68,82,83,84,85,86]. The suppression of multiple-scattered light is demonstrated with full-field imaging configurations only, which project the conjugate of the objective focal plane to a scientific camera [82,83,84,85]. As a consequence, in full-field OCT (FF-OCT) which does not require beam scanning, the spatial frequency spectrum or the angle-resolved image of the field which is backscattered from the sample is observed. Complex-valued signal acquisition is enabled through interferometric techniques such as phase-shifting interferometry [82,83] or off-axis holography [84,85].

Kang et al. demonstrated the acquisition of the time-gated reflection matrix with a set of plane wave basis modes incident on the sample at different angles, respectively [85]. The reflection matrix corresponds to the complex-valued backscattered field which is captured in case of sample illumination with the individual modes and, thus, quantifies the angle-resolved backscattered field depending on the angle of illumination. The reflection matrix contains contributions from single-scattered and from multiple-scattered light. In case of single scattering at an object which is placed inside the turbid layer, however, the change in lateral momentum of the backscattered wave reflects the spatial frequency of the hidden object, which is the object’s transfer function [85]. Depth-enhanced OCT imaging is achieved by collective accumulation of single scattering (CASS). Reflection matrix elements with equal momentum difference between the incident and the backscattered wave correspond to the same component of the object transfer function and match in phase. In contrast, the phase of signal contributions from multiple-scattered light is randomly distributed. Hence, the summation of complex-valued reflection matrix elements with equal momentum difference enhances single-scattered signal contributions compared to the contributions from multiple-scattered light and yields the object transfer function, from which the Fourier-transformed image of the hidden object is received.

The approach was experimentally demonstrated to be feasible for the micrometer resolution full-field imaging of samples hidden below tissue samples up to 0.9 mm thick [85]. Recently, an improved implementation of the technique was demonstrated which utilizes random phase patterns for the acquisition of the reflection matrix instead of a plane wave basis [84]. To further enhance the penetration depth, optical aberrations present in the sample are identified and corrected for in post-processing. The approach, in principle, applies an additional digital phase-correction to the experimentally acquired reflection matrix which is iteratively optimized such that the total intensity of the CASS image becomes maximal. The final optimized phase map reflects aberrations which are present in the sample.

Badon et al. demonstrated the suppression of multiple-scattered light based on spatial correlations of the incident and the backscattered field [82]. The group utilized an SLM to scan the position of point illumination at the sample and acquired the respective full-field image by Fourier transforming the angle-resolved backscattered field, which is captured with the experimental device. The reflection matrix, thus, yields the spatial distribution of the backscattered field depending on the point of illumination. In principle, a wide field OCT image with confocal illumination and detection can be constructed from the diagonal elements of the matrix. This technique is equivalent to conventional FF-OCT imaging relying on en face illumination. In another approach, the OCT signal corresponding to single-scattered light is expected to be detected close to the position of illumination, i.e., near the diagonal elements of the reflection matrix. Off-diagonal elements hence are removed to suppress signal contributions from multiple-scattered light [82]. A subsequent singular-value decomposition of the filtered matrix allows one to identify strongly reflecting particles embedded to the sample, similar to the techniques discussed in Section 2.4.3. The approach was experimentally demonstrated to enable micrometer resolution imaging of objects hidden below biological tissue up to 0.8 mm thick.

Very recently, Badon et al. demonstrated an enhanced post-processing method based on the distortion matrix [90]. This matrix describes the difference of the reflection matrix which is acquired with point-by-point sample illumination to the signal which is expected in case the beam is reflected at an ideal point-source in the sample plane and back-propagated through a homogeneous non-scattering medium. A singular value decomposition of the distortion matrix additionally allows one to identify and correct for optical aberrations present in the sample, similar to the iterative approach presented by Kang et al. [84], and enables imaging [90].

### 3.3. Spectral and Temporal Shaping of Scattered Light

The discussion on wavefront shaping and transmission matrix approaches which is given in Section 2 considered monochromatic radiation and manipulation and detection of the optical wavefront in the spatial domain only. For OCT imaging, broadband light sources are required, however, and the signal is captured time-of-flight dependent, i.e., in the temporal or spectral domain. This section illustrates general aspects of wavefront shaping with broadband sources and discusses temporal and spectral shaping of scattered light based on spatial manipulation of the beam which is incident on the turbid sample.

A number of reports demonstrated iterative wavefront shaping by probing the sample’s non-linear optical response, for example, the emitted two photon fluorescence, and optimizing the incident wavefront such that this signal is maximized (Section 2.3.2) [48,49,50,51,52,53,54,55,56]. To this end, Ti:Sapphire sources were used, which can be employed for OCT imaging as well, and spatial focusing of scattered light was demonstrated experimentally with the pulsed sources. To maximize the sample’s non-linear optical response, the pulsed illumination needs not only to be spatially focused but temporally focused as well to yield a high instantaneous intensity at the fluorophore, however [57]. As a consequence, iterative wavefront shaping with a non-linear optical feedback probe results in spatial and temporal compression of the scattered laser pulse. The effect was directly observed by Katz et al. in 2011.

Typical phase-only SLMs which are used for wavefront shaping experiments allow one to (axially) shift the modulated beam by only one to two wavelengths, which is not sufficient to significantly change the temporal profile of the beam. Fortunately, the turbid medium couples the spatial profile of the incident beam to the temporal and spectral shape of the scattered field. The effect can be understood by considering the scattering medium to be a linear and time invariant system. Adopting the model given in Section 2.3 and Figure 5, transmission from the n-th element of the incident wavefront to the m-th element of the scattered beam can be described based on the sample’s impulse response $h_{mn}\left(t\right)$:

(11) $$E_{m}^{det}\left(t\right)=\;\overset{N}{\underset{n=1}{\sum }}h_{mn}\left(t\right)\;⊛E_{n}^{src}\left(t\right)$$

In contrast, the optical transmission matrix $t_{mn}$ describes the propagation of monochromatic radiation only (Equation (2)). The symbol ⊛ denotes the convolution operator. The approach is equivalent to descriptions based on the sample’s Green function [55,91].

**Figure 10:**
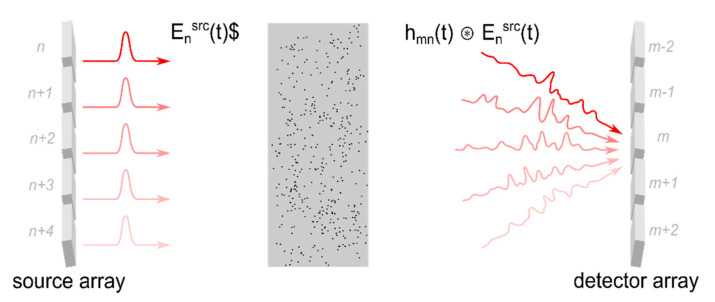
Coupling of temporal and spatial degrees of freedom in a scattering medium. Illuminating a sample with varying spatial modes causes different spatial (not shown, see Figure 5) and temporal speckle profiles to be observed behind a turbid sample. Spatial shaping of the incident beam enables temporal manipulation of the scattered field. Image from [8], with permission.

Each segment of the incident beam or, more generally, each incident mode $E_{n}^{src}\left(t\right)$ gives rise to a different spatial and temporal field distribution $h_{mn}\left(t\right)⊛E_{n}^{src}\left(t\right)$ in the detection plane [57,92]. The effect is illustrated in Figure 10 and can be understood by taking into account that the individual incident modes couple to different areas of the scattering sample. Thus, different temporal and spatial profiles result depending on the respective trajectory of the scattered field.

Spatial manipulation of the beam incident on the sample yields control over the temporal profile after scattering. For example, light can selectively be coupled to trajectories which give rise to the same temporal delay. In another approach, scattered light can be spatially and temporally focused by manipulating the spatial phase profile of the incident beam without actually manipulating the temporal profile. Due to the linearity of propagation, phase manipulation of the respective incident modes shifts the corresponding contributions to the scattered field accordingly, compare Figure 6. The phase profile of the incident beam, thus, can be optimized such that constructive interference is created from scattered light at one point in space and time, similar to the approaches which are discussed in Section 2 for monochromatic radiation.

Aulbach et al. initially demonstrated shaping and compression of a short laser pulse based on iterative wavefront optimization in 2011 [93]. The group superimposed the scattered pulse with a static reference beam at a point detector placed behind the turbid sample. The approach selectively detects light which is scattered to the position of the detector at the temporal delay which matches the temporal delay of the reference beam. Spatial and temporal focusing at the detector was demonstrated with an iterative algorithm which optimizes the phase profile of the incident beam such that the detector signal is maximized. In a similar approach, Mounaix et al. demonstrated the full field acquisition of the scattered field after sample illumination with a pulsed source. The technique allows one to determine the transmission matrix from light whose path length matches the length of the reference beam only, i.e., which is detected at a given temporal delay [94]. The approach hence is similar to the acquisition of the time-gated reflection matrix, which is discussed in Section 3.4, but determines the scattered field in transmission geometry. Phase conjugation based on the time-gated transmission matrix was shown to enable spatial and temporal focusing of scattered light [94,95] (compare Section 2.4.2).

A turbid medium couples the spatial profile of the incident beam to the spectrum of scattered light, as well. Fourier transforming Equation (11) yields [91,96,97,98,99]:

(12) $$E_{m}^{det}\left(\omega \right)=\;\overset{N}{\underset{n=1}{\sum }}h_{mn}\left(\omega \right)E_{n}^{src}\left(\omega \right)$$

As with the temporal profile, each mode $E_{n}^{src}\left(\omega \right)$ which is incident on the sample gives rise to a different spatial and spectral profile $h_{mn}\left(\omega \right)E_{n}^{src}\left(\omega \right)$ at the detection plane [57,91,92,97,99]. Hence, spectral shaping of the scattered field is enabled through spatial manipulation of the beam incident on the sample as well, similar to temporal shaping [92,99,100].

Equation (12) is equivalent to describing the scattered field in terms of the spectrally resolved transmission matrix $t_{mn}\left(\omega \right)$ which can be determined experimentally with a monochromatic source tuned to different wavelengths, for example [96,97]. Knowledge of the spectrally resolved transmission matrix was shown to yield spatial control over the scattered field, similar to the monochromatic transmission matrix, Section 2.4, and temporal or spectral control, as well [95,96,97].

### 3.4. Wavefront Shaping Techniques for Direct OCT Signal Enhancement

#### 3.4.1. Technical Implementation

The combination of wavefront shaping and OCT was first demonstrated in 2012 by Reto Fiolka et al. [70]. The group included a deformable mirror to the sample arm of a TD-OCT system, Figure 9b, and implemented Cui’s parallelized iterative wavefront shaping algorithm to optimize the amplitude of the OCT signal which is received from a scattering sample [67,70]. The combination with SD-OCT was demonstrated by Jang et al. [71] and by Choi et al. [68] in 2013. Both groups implemented algorithms which are similar to transmission matrix approaches, as explained in Section 2.4.

Jang et al. included a DMD to the source beam of an SD-OCT system [71] (Figure 9c). The DMD enables high-speed wavefront manipulation and the optical design allows one to feed the shaped wavefront to the scan head of a conventional SD-OCT system, which includes the optical elements of the reference and sample beam and which enables high-speed object scanning. On the other hand, the DMD enables binary amplitude wavefront manipulation only. To overcome this problem, Jang et al. noted that the phase of the beam which is diffracted at the DMD can be manipulated by laterally shifting the amplitude pattern which is applied to the device. The group, thus, implemented a wavefront shaping algorithm which uses the DMD to sequentially create different basis modes and which captures the OCT signal for 25 different lateral positions of the respective amplitude pattern at the DMD screen. A wavefront which enhances the OCT signal received at an arbitrarily chosen time-of-flight is then calculated. For each basis mode, the lateral position of the corresponding amplitude pattern is chosen which yields the highest signal amplitude at the target. The phase of the OCT signal is neglected. Subsequently, the laterally shifted patterns from all modes are superimposed and applied to the DMD. Similar to transmission matrix approaches, a wavefront which enhances the signal at any position within the axial FOV of the OCT system can be found without further signal acquisition. In contrast, however, the approach is purely based on the intensity of the acquired OCT signal. The phase of the incident field is not directly manipulated and the phase of the scattered field cannot be detected since the reference beam does not remain static in case different wavefronts are applied due to the optical design, Figure 9c.

In contrast, Choi et al. presented a Mach–Zehnder-based SD-OCT design with a phase-only SLM placed at the sample beam, Figure 9a [68]. The design features a static reference beam and enables the acquisition of the complex-valued SD-OCT signal from just a single acquisition of the spectral raw data. The group utilized the SLM to apply a set of different basis modes to the sample beam. The resulting SD-OCT signal corresponds to the time-of-flight resolved backscattered field, respectively. Similar to the acquisition of the optical transmission matrix, Section 2.4.1, the time-of-flight resolved reflection matrix, hence, can be acquired by iterating the respective basis modes and saving the resulting complex-valued OCT signal to the corresponding column of the matrix [68]. The total number of measurements which are required to capture the full reflection matrix, thus, is at least three times lower compared to phase-shifting algorithms (Section 2.4.1) and matches the number N of incident modes exactly. The group further demonstrated a phase conjugation algorithm equivalent to the approach discussed in Section 2.4.2 to be able to calculate an optimized wavefront from the reflection matrix which selectively enhances the received SD-OCT signal once the wavefront is applied to the sample beam. Phase conjugation with the time-resolved reflection matrix, hence, yields similar results to the iterative optimization algorithm which was demonstrated by Jang et al. [71]. A significantly reduced number of signal acquisitions is required. On the other hand, the approach necessitates a static reference beam to capture the reflection matrix and phase-only modulation to apply the optimized wavefront.

**Figure 11:**
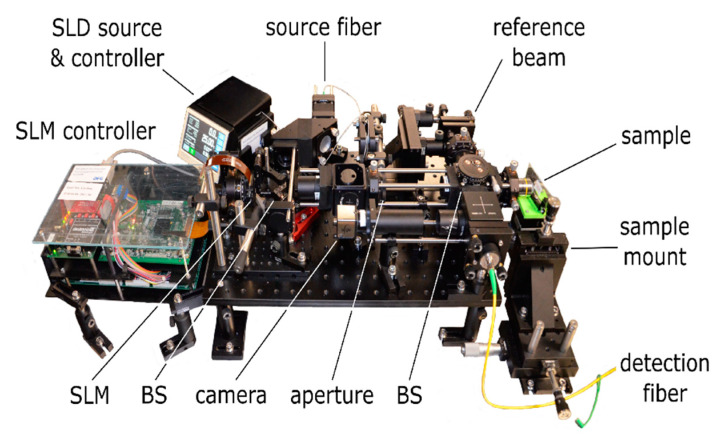
Photograph of a modular setup for independent wavefront manipulation at the sample and reference arm of a spectral domain OCT device. Abbreviations: SLM—spatial light modulator, BS—beam splitter. Image from [8], with permission.

**Figure 12:**
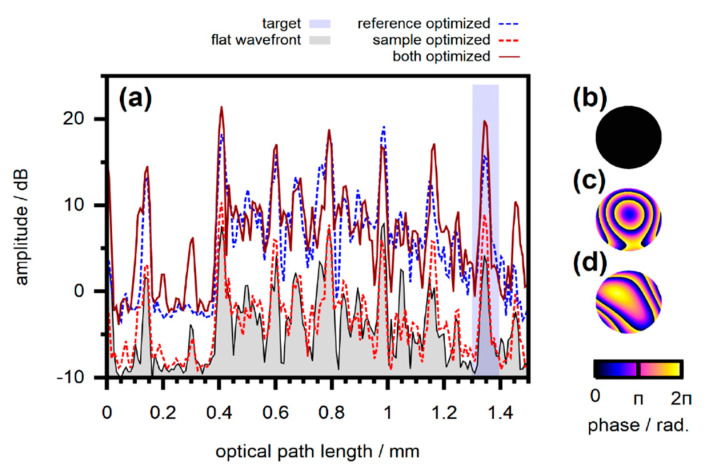
Adaptive optics correction and enhancement of OCT signal. (**a**) OCT A-scans taken at the sample with different phase patterns applied. The depth-range from which the feedback signal for wavefront optimization is calculated is highlighted (light blue area). (**b**) Flat wavefront applied to both beams for the acquisition of the initial scan (signal marked by grey area). The pattern applied corresponds to a beam diameter of 4 mm at the SLM. (**c**,**d**) Wavefronts optimized at the sample beam and reference beam, respectively. Image adapted from [8,101], with permission. The signal enhancement obtained is clearly visible.

Kanngiesser et al. reported a technique for independent wavefront manipulation at the sample and reference arm of a spectral domain OCT device [101]. The approach is based on a single spatial light modulator and can be implemented to existing free space SD-OCT systems through the introduction of an additional interferometer at the light source; see Figure 11 for a modular setup realized in the laboratory [102]. An example for typical results obtained by employing this setup is shown Figure 12 where adaptive optics correction and enhancement of OCT signals from self-made opaque OCT phantoms is demonstrated. The samples were created from multiple layers of scattering polymer film (Parafilm M, Pechiney Plastic Packaging, Chicago, IL, USA) in between a glass objective slide and cover glass. Independent single-pass wavefront manipulation and beam shaping at either arm of the interferometer is possible, in case a phase-only spatial light modulator is used, for independent phase manipulation. The system was also employed to demonstrate complex-valued OCT signal acquisition by phase shifting combined with iterative optical wavefront shaping. This leads to local enhancement of the OCT signal acquired from a scattering sample and is intended for use in strongly scattering media in future. The design is highly versatile and allows for digital switching between applications by changing the pattern applied to the SLM only.

#### 3.4.2. Non-Invasive Focusing Approaches

OCT signal enhancement based on wavefront shaping, in principle, focuses back-scattered light at a given time-of-flight to the position of the imaging system’s detector [68], similar to the experiments in transmission geometry reported by Aulbach et al. [93]; see Section 3.3. If the technique is supposed to be used for depth-enhanced imaging, it is important to investigate in which fashion the approach affects the light distribution inside the scattering sample. Ideally, one wishes the detected OCT signal to be proportional to the electric field which is single scattered at the object to be imaged. Using wavefront shaping to maximize the OCT signal in this case enhances the intensity at the detection volume, i.e., at the object, and enables non-invasive focusing. With practical systems, these ideal conditions cannot be met, however, since OCT devices detect multiple-scattered light to some extent, as well. Even in the presence of multiple-scattered light, the amplitude and the SNR of the OCT signal can be enhanced in case light is focused to the position of the hidden object though.

Fiolka et al. demonstrated non-invasive optical focusing by embedding small reflecting particles behind a forward scattering turbid layer and using an iterative wavefront shaping algorithm to enhance the OCT signal which is detected from these particles [70]. The technique requires the target particles to be sparsely distributed to ensure the detected signal corresponds to the reflection at a single particle only. In case multiple particles are simultaneously present at the detection volume, the approach is observed to produce a split focus [103] since the OCT signal is proportional to the field reflected from either particle. The technique further requires to clearly identify the OCT signal which is resulting from the respective target particles, i.e., the particles need to be visible in the OCT scan. For depth-enhanced imaging, one actually is interested in the case conventional OCT imaging is not possible, though, i.e., signal contributions from multiple-scattered light dominate compared to weakly scattered light.

Jeong et al. demonstrated non-invasive focusing inside a scattering sample based on a singular value decomposition of the time-gated reflection matrix [86] (compare Section 3.2). In principle, the largest eigenvalue and the corresponding eigenvector of the matrix reflect the wavefront with the highest intensity after reflection at the sample. In case a strongly reflecting target object is hidden inside the turbid medium, this wavefront was shown to preferentially couple light to trajectories which interact with the target compared to trajectories without any target interaction [86]. In terms of OCT imaging, the former signal contributions reflect the sample morphology, whereas the latter are considered signal noise from multiple-scattered light. The effect becomes stronger the smaller the object gets and was demonstrated to enable non-invasive focusing at the hidden target.

Choi et al. demonstrated the acquisition and singular value decomposition of the reflection matrix with a monochromatic source and compared the approach to iterative wavefront shaping based on the backscattered field [104]. The group demonstrated the iterative wavefront shaping algorithm to preferentially couple light to those eigenmodes of the scattering sample which correspond to the largest eigenvalues. The experiment was recently repeated with time-gated acquisition similar to OCT [105]. Due to the preferential coupling to high-reflectivity eigenmodes, iterative wavefront shaping based on the OCT signal was demonstrated to focus light to a strongly reflecting target embedded to the sample as well, similar to the previous approach based on the singular value decomposition of the time-gated reflection matrix [86].

The previous reports considered a partitioning algorithm (compare [66]) for iterative wavefront optimization [104,105]. Other iterative optimization algorithms are expected to find comparable wavefronts, and iterative wavefront shaping further is equivalent to point-wise focusing based on phase conjugation with the reflection matrix, Section 2.4.2. Hence, no matter what kind of wavefront optimization procedure is utilized, light which is scattered at strongly reflecting sample structures and the corresponding contributions to the OCT signal are expected to be predominantly enhanced with wavefront shaping, even in case these signal features cannot clearly be identified in the original OCT signal due to multiple scattering.

#### 3.4.3. Depth-Enhanced Imaging

**Figure 13:**
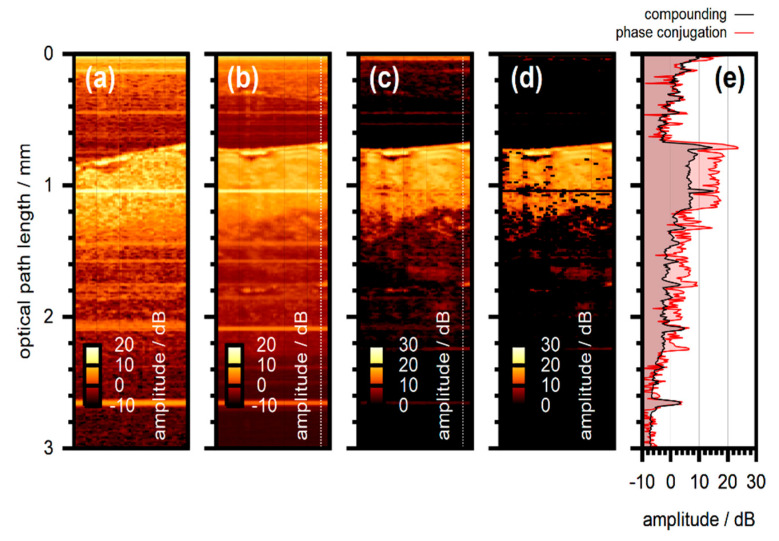
Wavefront shaping at layered scattering phantom. Phase conjugation with biological tissue. (**a**) Conventional OCT scan. (**b**) Image captured with a speckle-compounding algorithm to reduce speckle contrast. (**c**) Scan acquired with phase conjugation. Number of modes N = 256. (**d**) Phase conjugation with additional artefact suppression. (**e**) Amplitude of the two A-scans marked by the dashed line in panels (**b**) and (**c**), respectively. The amplitude color-scale corresponds to the same dynamic range of 30 dB for all scans. Adapted from [8,106], with permission.

Kanngiesser and Roth presented an analysis on how the time-resolved reflection matrix relates to the SD-OCT signal [106]. It was demonstrated theoretically as well as experimentally that phase conjugation with the matrix enhances the OCT signal depth-selectively, but not image artefacts. The applicability for imaging of scattering media was achieved. As an effect of the phase conjugation applied the signal-to-noise ratio (SNR) could be increased and the speckle contrast reduced. The approach was also applied to selected biological samples, i.e., a sample cut from a food-quality chicken thigh; see Figure 13. The conventional OCT signal, Figure 13a, is subject to strong speckles which appear rather coarse due to the large lateral step width chosen to be 20 µm, in this case. As a consequence, the lower boundary of the epidermis is not clearly visible, even though avian skin is rather thin compared to that of mammals, and the tissue morphology is not evident from the OCT signal. With a so-called speckle-compounding algorithm applied to reduce the speckle contrast by averaging the OCT signal over multiple independent realizations of the speckle pattern (Figure 13b), speckles are reduced and the boundary between the epidermis and the dermis becomes visible at a depth of about 1.1 mm as well as some structures which are located deeper in the dermis. The epidermis produces a stronger OCT signal and, hence, the position of the dermal-epidermal junction is also evident from the single A-scan illustrated in Panel (e). Figure 13c shows the image which is captured with the phase conjugation algorithm. To better estimate the SNR, the amplitude color-scale is chosen to cover the same dynamic range of 30 dB which is used for panels (a) and (b), as well. With the phase conjugation algorithm, the signal amplitude received from backscattering sample structures such as the epidermis is enhanced; see again Panel (e). An increased image contrast compared to the compounding algorithm (Figure 13b) and, hence, a better SNR are observed. Furthermore, considering Figure 13c, the phase conjugation algorithm is found to reduce the speckle contrast similar to the compounding algorithm. Speckles result from interference of uncontrolled waves which are randomly backscattered to the detector. The phase conjugation algorithm stitches the OCT image from a set of scans which are optimized for signal enhancement, i.e., for constructive interference of the backscattered field, at each voxel of the OCT scan individually. The image which is received with the phase conjugation algorithm, hence, can be considered to be constructed from bright speckles only. Figure 13d demonstrates signal enhancement with additional artefact suppression applied. The amplitude of two selected A-scans marked by dashed lines in the respective panels is given in Figure 13e. In future, the approach presented is intended to be further developed for in vivo imaging.

Imaging based on the direct enhancement of the OCT signal through wavefront shaping approaches, in contrast to the techniques discussed in Section 3.2, is also demonstrated, for example, by the group of Park with the system which was presented by Jang et al. in 2013; compare Section 3.4.1 [71,87,88,89]. Iterative wavefront shaping is utilized to selectively enhance the amplitude of the SD-OCT signal at a given time-of-flight one at a time. Depth-enhanced imaging is enabled by optimizing the incident wavefront for signal enhancement at different positions in the axial FOV individually and by stitching a full depth-scan from the in-target point-optimized OCT signals [71].

The acquisition time required to capture a single A-scan is determined by the time required to find the optimized wavefronts, similar to the acquisition of the reflection matrix, and by the time which is required to subsequently scan the axial position of point-wise signal enhancement for imaging. The former is determined by the optimization algorithm, whereas the latter corresponds to the pixel count at the axial FOV of the optimized A-scan. In contrast to comparable wavefront optimization algorithms, the method proposed by Jang et al. requires a rather high number of acquisitions (Section 3.4.1). Due to high-speed wavefront manipulation enabled with a DMD and due to efficient data processing, the optimization of a single depth-scan could be demonstrated within 15 s for a set of 300 basis modes and for 200 pixels at the optimized A-scan, nonetheless [87]. Cross-sectional imaging is enabled by scanning the position of sample illumination and repeating the full optimization process at each lateral position, respectively. The approach was shown to enable depth-enhanced SD-OCT imaging with biological samples [87,88,89] and to be sufficiently fast for in-vivo imaging with anaesthetized and fixated mice [87].

## 4. Remaining Problems

A number of approaches demonstrated depth-enhanced FF-OCT imaging based on the acquisition of the time-gated optical reflection matrix (Section 3.2). The technique, in principle, exploits correlations between the incident and the backscattered sample beam to additionally suppress multiple-scattered light which is detected by the OCT system. The acquisition of the time-gated reflection matrix requires sophisticated optical designs which cannot easily be implemented with existing OCT devices. Furthermore, the approach was demonstrated with phase-only liquid crystal SLMs only, which are subject to low frame rates and, hence, cause long acquisition times which prohibit in-vivo imaging.

The group of Park demonstrated depth-enhanced OCT imaging based on iterative wavefront shaping (Section 3.4.3). The presented system can easily be implemented by modifying existing SD-OCT systems to include a DMD for wavefront manipulation at the source beam, Figure 9c. The approach potentially enables high-speed imaging since fast SLM as well as commercial SD-OCT heads, which include beam scanning optics, can be used. The experimentally demonstrated system still required 15 s to capture a single A-scan, however. This number is too high for in-vivo imaging applications with most biological samples and is mainly caused by the inefficient optimization algorithm, which requires a large number of iterations.

In contrast, Choi et al. demonstrated a potential high-speed algorithm which enables OCT signal enhancement based on the time-resolved reflection matrix. The number of acquisitions required to capture the matrix is reduced 25-fold compared to the algorithm presented by Jang et al. (Section 3.4.1). On the other hand, the approach requires a sophisticated optical design, since the reference beam is necessary to remain static during the acquisition of the reflection matrix, and phase-only modulation of the beam which is incident on the sample. As a consequence, the approach cannot be implemented by modifying existing SD-OCT systems and the utilization of fast micro-electro-mechanical systems for wavefront manipulation is not yet demonstrated. One column of the time-resolved reflection matrix is taken from a single acquisition of the complex-valued SD-OCT signal. The matrix, in principle, is supposed to reflect the complex-valued backscattered field which results from sample illumination with the respective basis modes. The mutual interference component of the complex SD-OCT signal is proportional to the field which is backscattered from the sample, but image artefacts which are additionally detected with SD-OCT systems are not. To date, a detailed theoretical investigation on how the time-resolved reflection matrix relates to the complex-valued SD-OCT signal is not yet reported and the impact of image artefacts on the acquisition of the matrix and on subsequent phase conjugation for OCT signal enhancement is not discussed.

Jang et al. and Choi et al. both demonstrated the amplitude of the OCT signal to be enhanced as an effect of iterative wavefront shaping or optical phase conjugation. Yu et al. demonstrated the iterative algorithm to enhance the penetration depth of OCT systems when imaging scattering media [89]. The group defined the penetration depth to correspond to that optical path length at which the amplitude of the enhanced OCT signal drops below the noise threshold of the imaging system. It was not investigated so far whether signal contributions from multiple-scattered light are enhanced by wavefront shaping as well, though, and thus it is not clear whether an actual benefit for imaging with turbid media exists. Imaging applications of phase conjugation with the time-resolved reflection matrix are not yet demonstrated at all.

## 5. Conclusions

In this article, we review the application of wavefront shaping techniques to optical coherence tomography. We show that OCT can strongly benefit from a number of different approaches for wavefront shaping which usually control the scattered light field by manipulating the field incident on the sample. The main advantages are the enhancement of the OCT signal and the increase in the penetration depth which in particular improves imaging in strongly scattering samples, e.g., biological tissue. We highlight that the applications such non-destructive testing, metrology and non-invasive medical diagnostics can strongly benefit from the approach and discuss the current limitations and challenges. As an example, OCT imaging enhanced by wavefront shaping could be beneficial for the realization of an optical biopsy for skin disease detection [107,108,109]. As such, concepts usually require multimodal optical measurement, OCT combined with wavefront shaping could be employed to uncover details of the morphology of suspicious skin lesions which are hidden beneath the surface. Once localized, these could then be investigated using further optical modalities, e.g., Raman spectroscopy, to assess the pathophysiology, i.e., whether the particular part of the lesion is malignant or benign. The fused measurement data from all modalities could then allow for more objective and non-invasive medical diagnostics. This example stands for a whole class of applications necessitating in vivo measurement at high speed, accuracy and resolution. Thus, the challenges to be addressed in future work include the increase in image acquisition speed and resolution as well as the miniaturization of the devices to facilitate real-world applications, e.g., in clinical environments.
